# Supplementary material for: Mining on Alzheimer’s diseases related knowledge graph to identity potential AD-related semantic triples for drug repurposing
Source: BMC Bioinformatics. 2022 Sep 30;23(Suppl 6):407. doi: 10.1186/s12859-022-04934-1 (PMC9523633; doi:10.1186/s12859-022-04934-1)
Supplement: Supplementary file 1 — Additional file 1. AD-related UMLS concepts used in the study. [file 12859_2022_4934_MOESM1_ESM.pdf]

Additional Files
Tables

Table 9 Special Subjects and Objects Been Kept

Object/Subject Name	CUI
Alzheimer Disease, Early Onset	C0750901
Alzheimer Disease, Late Onset	C0494463
Focal Alzheimer's disease	C0338450
Familial Alzheimer's disease	C0276496
Alzheimer's disease treatment	C1979617
Alzheimer's disease antigen	C0051532
Alzheimer's Disease	C0002395
Alzheimer Disease 11	C1853360
Alzheimer Disease 14	C1970144
Alzheimer Disease 16	C2677888
Alzheimer Disease 7	C1853555
Alzheimer Disease 8	C1846735
Alzheimer disease, familial, type 3	C1843013
Alzheimer Vaccines	C0949574
